# Clinical Trial Discussion and Participation in a Breast Cancer Cohort by Race and Ethnicity

**DOI:** 10.1001/jamanetworkopen.2025.15205

**Published:** 2025-06-12

**Authors:** Nan Chen, Jincong Q. Freeman, Fangyuan Zhao, Leah Goldberg, Sudha R. Yarlagadda, Elizabeth Terman, Dezheng Huo, Rita Nanda

**Affiliations:** 1 Section of Hematology/Oncology, Department of Medicine, University of Chicago, Chicago, Illinois; 2 Department of Public Health Sciences, University of Chicago, Chicago, Illinois; 3 Center for Health and the Social Sciences, University of Chicago, Chicago, Illinois; 4 Department of Medicine, University of Chicago, Chicago, Illinois; 5 Center for Innovation in Global Health, University of Chicago, Chicago, Illinois; 6 Now with Key Laboratory of Carcinogenesis and Translational Research, Peking University Cancer Hospital & Institute, Beijing, China.

## Abstract

**Importance:**

Racial and ethnic disparities in breast cancer clinical trial participation pose a significant barrier to providing equitable care. Black and Hispanic patients are underrepresented in clinical trials, and an improved understanding of barriers to enrollment is needed.

**Objective:**

To examine patterns of clinical trial discussion and participation and patient attitudes toward clinical trial participation in a diverse cohort of patients with breast cancer.

**Design, Setting, and Participants:**

This cross-sectional study used survey data from patients enrolled in the Chicago Multiethnic Epidemiologic Breast Cancer Cohort. Patients were queried about clinical trial discussion and subsequent enrollment in a therapeutic clinical trial. Barriers to trial enrollment were also assessed. Surveys were conducted from July to September 2022, and data were analyzed from February to October 2024.

**Exposure:**

Self-reported race and ethnicity, including Asian, Black, Hispanic, and White.

**Main Outcomes and Measures:**

Outcomes of interest were discussing participation in a breast cancer clinical trial with a health care practitioner, participating in a clinical trial, and barriers to trial enrollment.

**Results:**

Of 1150 respondents (mean [SD] age, 53.7 [11.9] years), 51 (4.4%) were Asian, 224 (19.5%) were Black, 35 (3.1%) were Hispanic, and 838 (73.0%) were White. A total of 447 respondents (38.9%) reported discussing trial participation with a health care practitioner. There were no differences in trial discussion between White patients and other racial groups (Asian: adjusted odds ratio [AOR], 0.75; 95% CI, 0.31-1.82; Black: AOR, 1.31; 95% CI, 0.78-2.21; Hispanic: AOR, 0.73; 95% CI, 0.26-2.08). Among 443 patients offered a trial, 285 (64.3%) participated. While there were differences in trial participation across racial and ethnic groups, these differences were not significant after adjusting for sociodemographic and clinical factors. Among 158 patients who did not enroll in the trial offered, 37 (23.4%) reported ineligibility, 17 (10.8%) were worried about the possibility of getting a placebo, 16 (10.1%) were worried about extra time required, and 14 (8.9%) were worried about possible adverse effects.

**Conclusions and Relevance:**

This cross-sectional study demonstrated that when offered, patients across racial and ethnic groups were equally likely to participate in clinical trials. In addition to ineligibility, time toxicity was a significant barrier to enrollment. These data provide valuable insights that can serve as a roadmap for how to expand access to trials for all patients, regardless of racial, ethnic, and socioeconomic background.

## Key Points

**Question:**

Do clinical trial discussion, participation, and patient attitudes toward enrollment differ by race and ethnicity in a diverse cohort of patients with breast cancer?

**Findings:**

This cross-sectional study of 1150 patients found that patients from different racial and ethnic backgrounds were equally likely to discuss and participate in clinical trials when offered. However, patients reported that concerns over the time commitment required was a barrier to trial enrollment.

**Meaning:**

These data provide valuable insights into the expansion of access to clinical trial enrollment to ensure inclusion of patients from underrepresented racial and ethnic groups; reducing barriers to enrollment of clinical trials is a scientific and ethical imperative for the cancer community.

## Teaser

This cross-sectional study examines patterns of discussion and participation in clinical trials as well as attitudes regarding participation in a racially and ethnically diverse cohort of patients with breast cancer.

## Introduction

Breast cancer is the second leading cause of cancer mortality among women in the US^1^; however, this burden disproportionately affects Black women, who have a 40% higher mortality compared with White women.^1^ Multiple factors contribute to this disparity, including a higher likelihood of diagnosis at later stages,^1^ higher rates of triple-negative breast cancer (TNBC), and greater delays in treatment initiation.^2,3^ Furthermore, socioeconomic factors are integral to addressing disparities in breast cancer mortality. Various studies have demonstrated local variations in breast cancer mortality,^4,5^ including an analysis of the Black Women’s Health Study using neighborhood-level data to show that disadvantaged neighborhoods are associated with higher rates of estrogen receptor–negative breast cancer.^5^ Although breast cancer survival has significantly improved due to advances in treatment, significant racial gaps in treatment receipt and mortality remain.

Clinical trials are an important part of breast cancer care, with opportunities for patients to receive novel therapies and oncologists to better understand therapies in diverse patient populations.^6^ However, enrollment among Black and Hispanic women is disproportionately low, resulting in study populations that fail to capture the racial and ethnic diversity of patients with breast cancer.^2,7^ Retrospective evaluation of racial and ethnic disparities in clinical trials is difficult due to underreporting of race and ethnicity.^8^ Nonetheless, a review of registrational trials leading to US Food and Drug Administration approvals from 2008 to 2018 across multiple cancer types found that Black patients were significantly underrepresented, constituting only 3.1% of total trial participants, compared with 14.1% of expected trial participants.^9^ Another study using a national cancer database reported that Black women and Hispanic women had significantly lower rates of participating in a breast cancer trial, with adjusted hazard ratios of 0.67 and 0.45, respectively, compared with White patients.^7^ Moreover, there was a decline in enrollment by more than 30% among Black women in oncology trials from 2003 to 2012 compared with 1996 to 2002.^8^

Addressing clinical trial enrollment disparities is an important strategy to reduce racial and ethnic inequities in breast cancer care.^10^ However, there is a lack of knowledge in patterns of discussion of trials with patients, patterns of participation in clinical trials, and reasons for choosing or declining to participate. It is imperative to better understand the barriers racial and ethnic minority women, such as Asian, Black, and Hispanic women, face in clinical trial participation, to enroll more diverse patient populations that reflect the true epidemiology of breast cancer. This single-center, survey-based study sought to evaluate patterns of clinical trial discussion and enrollment and to describe patient attitudes toward clinical trial participation in a diverse cohort of patients with breast cancer.

## Methods

### Design, Setting, and Participants

This was a cross-sectional survey study analyzing data collected from patients with breast cancer enrolled in the Chicago Multiethnic Epidemiologic Breast Cancer Cohort (ChiMEC) study. The University of Chicago institutional review board reviewed and approved this study. All participants provided a written consent to the ChiMEC study and follow-up surveys. We followed the Strengthening the Reporting of Observational Studies in Epidemiology (STROBE) reporting guideline. Detailed information of the ChiMEC study has been previously described.^11^ Briefly, ChiMEC is a hospital-based cohort established at the University of Chicago in 1993, and the number of patients enrolled in the cohort has accelerated since 2008. From July to September 2022, we sent a total of 1868 questionnaires to ChiMEC participants who consented to receive follow-up surveys. The mean (SD) duration between ChiMEC enrollment and the survey was 6.7 (4.0) years. The overall response rate was 66.2% (1236 participants) and of these, 1150 patients (93.0%) responded to the questions about clinical trial discussion and participation.

### Measures

One outcome of interest was clinical trial discussion. Participants were asked whether or not a breast cancer treatment clinical trial was discussed during their visit at the University of Chicago Medical Center. During the period of ChiMEC enrollment, multiple breast cancer clinical trials were available for participation. Another outcome of interest was clinical trial enrollment, which was measured by asking patients whether they participated in the clinical trials offered them at our hospital. Both outcome variables were dichotomous as yes and no. Participants were also asked whether they sought care with the intent to participate in a clinical trial at our cancer center. Furthermore, we assessed barriers or facilitators by asking patients the reasons they did or did not enroll in the clinical trials offered.

The main independent variable of interest was race and ethnicity. Racial and ethnic information was per patient self-report. Patients reported their primary race and Hispanic origin separately. In this study, we further categorized race and ethnicity into 4 groups: Hispanic, non-Hispanic Asian (hereafter, Asian), non-Hispanic Black (hereafter, Black), and non-Hispanic White (hereafter, White). Other demographic and clinical characteristics included age at diagnosis, age group (<40, 40-65, and >65 years), age at survey, duration between ChiMEC enrollment and survey, highest level of education (high school or less, trade or technical school or some college, Associate’s degree, Bachelor’s degree, and graduate or professional degree), marital status (married, single or not married, divorced, separated, and widowed), type of health insurance (private, Medicaid, Medicare, and other or unknown), annual household income, distance from residence to hospital, Area Deprivation Index (ADI), Charlson comorbidity index, histologic type (ductal, lobular, both ductal and lobular, other), American Joint Committee on Cancer (AJCC) stage group, molecular subtype (hormone receptor–positive and human epidermal growth factor 2–negative [ERBB2-negative], ERBB2-positive, and TNBC), and tumor grade. Distance from residence to hospital was calculated by taking the differences of coordinates (ie, longitudes and latitudes) between the patient’s address and UChicago Medicine Comprehensive Cancer Center’s address using the Haversine formula. The ADI is a composite measure using domains of income, education, employment, and housing quality, which ranks neighborhoods by socioeconomic disadvantage at the national level.^12^ The total scores range from 1 to 100, with higher scores representing greater neighborhood socioeconomic disadvantage. We further categorized ADI into 4 quartiles. The first quartile reflected the least socioeconomically deprived neighborhoods, while the fourth quartile reflected the most deprived neighborhoods.

### Statistical Analysis

We first described the patient cohort overall and by race and ethnicity using summary statistics. Specifically, means and SDs were calculate for continuous variables and compared using *t* tests, analysis of variance, Wilcoxon rank-sum, or Kruskal-Wallis tests. For categorical data, we tabulated frequencies and proportions and used Person χ^2^ or Fisher exact tests to compare the distributions across racial and ethnic groups. To examine racial and ethnic differences in clinical trial discussion or participation, separate multivariable logistic regression models were fit to calculate adjusted odds ratios (AORs) and the corresponding 95% CIs. We implemented the stepwise modeling approach. Model 1 contained AJCC stage group and molecular subtype. In addition to AJCC stage and molecular subtype, model 2 further included age at diagnosis, highest level of education, marital status, type of health insurance, annual household income, and Charlson comorbidity index. In the sensitivity analysis, we further controlled for distance from residence to hospital and ADI in regression models and observed similar results. The level of significance was set at *P* ≤ .05, and all statistical tests were 2-sided. All data analyses were performed using Stata software version 17 (StataCorp) from February to October 2024.

## Results

### Patient Characteristics

Of 1150 respondents included in our analysis, the overall mean (SD) age at diagnosis was 53.7 (11.9) years; 51 patients (4.4%) were Asian, 224 patients (19.5%) were Black, 35 patients (3.1%) were Hispanic, and 838 patients (73.0%) were White. Demographic, clinicopathologic, and socioeconomic data stratified by race and ethnicity are described in Table 1. The mean age of patients differed significantly between the 4 racial and ethnic groups. Black patients had higher percentages of public insurance coverage (Medicaid: 36 patients [16.1%] vs 10 patients [1.2%]; *P* < .001), were less likely to be married (79 patients [40.5%] vs 604 patients [78.7%]; *P* < .001), and had lower annual household income compared with White patients (Table 1). Additionally, higher proportions of Black patients were residing in more socioeconomically disadvantaged neighborhoods (both third and fourth quartiles of ADI) than other racial and ethnic groups (Table 1). Clinicopathologic differences among the 4 groups demonstrated significantly increased proportions of TNBC in Black patients (40 patients [25.0%]) than in Asian (5 patients [12.8%]), Hispanic (4 patients [14.3%]), or White (92 patients [14.6%]) patients (*P* < .001) and of grade 3 tumors (111 patients [55.5%]) in Black patients than in Asian (18 patients [38.3%]), Hispanic (13 patients [39.4%]), or White (276 patients [36.9%]) patients (*P* < .001). There were no differences in the proportion of patients within each group who came to our center specifically to participate in a clinical trial (Table 1).

**Table 1.** Demographic and Clinical Characteristics of Patients With Breast Cancer, Overall and by Race and Ethnicity

Characteristic	Patients, No. (%)					*P* value^a^
	Overall (N = 1150)	Asian (n = 51 [4.4%])	Black (n = 224 [19.5%])	Hispanic (n = 35 [3.1%])	White (n = 838 [73.0%])	
Age at survey, mean (SD), y	61.7 (12.0)	53.9 (10.7)	63.1 (12.7)	54.1 (11.5)	62.2 (11.6)	<.001
Age at diagnosis, y						
Mean (SD)	53.7 (11.9)	46.4 (10.8)	54.6 (12.9)	48.1 (12.2)	54.1 (11.5)	<.001
<40	154 (13.9)	13 (26.5)	29 (13.5)	9 (25.7)	103 (12.7)	.02
40-65	756 (68.0)	33 (67.3)	147 (68.4)	20 (57.1)	556 (68.5)	
>65	202 (18.2)	3 (6.1)	39 (18.1)	6 (17.1)	153 (18.8)	
Duration between diagnosis and survey, median (IQR), y	6.5 (3.6-10.9)	6.7 (4.5-9.1)	6.9 (3.7-11.6)	5.2 (2.0-8.0)	6.4 (3.6-11.0)	.08
Duration between ChiMEC enrollment and survey, y						
Median (IQR)	6.2 (3.4-10.0)	6.6 (4.3-9.0)	6.7 (3.4-11.1)	5.4 (2.4-8.2)	6.0 (3.3-9.9)	.17
<5	451 (39.3)	16 (31.4)	86 (38.4)	16 (45.7)	333 (39.7)	.55
≥5	697 (60.1)	35 (68.6)	138 (61.6)	19 (54.3)	505 (60.3)	
Highest level of education						
≤High school/GED	113 (9.8)	0	30 (13.4)	6 (17.1)	77 (9.2)	<.001
Trade/technical school, or some college	184 (16.0)	0	62 (27.7)	6 (17.1)	116 (13.9)	
Associate’s degree	71 (6.2)	2 (3.9)	11 (4.9)	3 (8.6)	55 (6.6)	
Bachelor’s degree	335 (29.2)	22 (43.1)	55 (24.6)	6 (17.1)	251 (30.0)	
Graduate or professional degree	446 (38.8)	27 (52.9)	66 (29.5)	14 (40.0)	338 (40.4)	
Marital status						
Married	738 (70.7)	33 (71.7)	79 (40.5)	22 (62.9)	604 (78.7)	<.001
Single or not married	192 (18.4)	9 (19.6)	81 (41.5)	10 (28.6)	92 (12.0)	
Divorced, separated, or widowed	114 (10.9)	4 (8.7)	35 (17.9)	3 (8.6)	71 (9.3)	
Type of health insurance						
Private	817 (71.0)	44 (86.3)	117 (52.2)	28 (80.0)	628 (74.9)	<.001
Medicaid	49 (4.3)	0	36 (16.1)	3 (8.6)	10 (1.2)	
Medicare	214 (18.6)	4 (7.8)	50 (22.3)	4 (11.4)	155 (18.5)	
Other or unknown	70 (6.1)	3 (5.9)	21 (9.4)	0	45 (5.4)	
Annual household income, $						
<50 000	116 (13.4)	3 (8.1)	55 (33.5)	9 (34.6)	49 (7.7)	<.001
50 000-74 999	94 (10.9)	2 (5.4)	30 (18.3)	1 (3.8)	61 (9.6)	
75 000-99 999	95 (11.0)	7 (18.9)	22 (13.4)	3 (11.5)	63 (9.9)	
100 000-149 999	131 (15.1)	10 (27.0)	14 (8.5)	1 (3.8)	106 (16.6)	
≥150 000	216 (24.9)	4 (10.8)	11 (6.7)	7 (26.9)	193 (30.3)	
Prefer not to answer	214 (24.7)	11 (29.7)	32 (19.5)	5 (19.2)	166 (26.0)	
Distance from residence to hospital, median (IQR), miles^b^	19.9 (9.6-32.4)	22.1 (10.2-33.3)	5.1 (2.9-16.8)	16.1 (7.7-27.9)	23.2 (13.6-34.6)	<.001
Area Deprivation Index, quartile^c^						
First	377 (34.4)	26 (53.1)	16 (7.8)	5 (14.7)	329 (40.8)	<.001
Second	373 (34.0)	14 (28.6)	39 (19.0)	20 (58.8)	299 (37.1)	
Third	249 (22.7)	6 (12.2)	95 (46.3)	5 (14.7)	143 (17.7)	
Fourth	97 (8.9)	3 (6.1)	55 (26.8)	4 (11.8)	35 (4.3)	
Charlson Comorbidity Index						
0	985 (88.6)	44 (89.8)	187 (87.0)	32 (91.4)	722 (88.9)	.53
1	62 (5.6)	1 (2.0)	14 (6.5)	3 (8.6)	43 (5.3)	
≥2	65 (5.9)	4 (8.2)	14 (6.5)	0	47 (5.8)	
Histologic type						
Ductal	736 (80.2)	34 (85.0)	145 (84.8)	25 (83.3)	531 (78.6)	.11
Lobular	92 (10.0)	2 (5.0)	11 (6.4)	1 (3.3)	78 (11.5)	
Ductal and lobular	54 (5.9)	4 (10.0)	9 (5.3)	4 (13.3)	37 (5.5)	
Other	36 (3.9)	0	6 (3.5)	0	30 (4.4)	
AJCC stage group						
0	196 (17.9)	9 (18.4)	45 (21.2)	5 (14.7)	137 (17.1)	.17
I	511 (46.6)	19 (38.8)	80 (37.7)	16 (47.1)	396 (49.4)	
II	268 (24.4)	15 (30.6)	60 (28.3)	10 (29.4)	182 (22.7)	
III	112 (10.2)	6 (12.2)	23 (10.8)	2 (5.9)	81 (10.1)	
IV	10 (0.9)	0	4 (1.9)	1 (2.9)	5 (0.6)	
Molecular subtype						
HR+/ERBB2−	568 (66.3)	21 (53.8)	83 (51.9)	20 (71.4)	444 (70.6)	<.001
ERBB2+	147 (17.2)	13 (33.3)	37 (23.1)	4 (14.3)	93 (14.8)	
TNBC	142 (16.6)	5 (12.8)	40 (25.0)	4 (14.3)	92 (14.6)	
Tumor grade						
1	146 (14.2)	6 (12.8)	12 (6.0)	4 (12.1)	124 (16.6)	<.001
2	464 (45.1)	23 (48.9)	77 (38.5)	16 (48.5)	348 (46.5)	
3	419 (40.7)	18 (38.3)	111 (55.5)	13 (39.4)	276 (36.9)	
Was your goal in coming to the University of Chicago to participate in a clinical trial?						
No	1084 (95.1)	47 (94.0)	206 (92.8)	33 (94.3)	796 (95.8)	.24
Yes	56 (4.9)	3 (6.0)	16 (7.2)	2 (5.7)	35 (4.2)	

Abbreviations: −, negative; +, positive; AJCC, American Joint Committee on Cancer; ChiMEC, Chicago Multiethnic Epidemiologic Breast Cancer Cohort; ERBB2, human epidermal growth factor receptor 2; GED, general educational development; HR, hormone receptor; TNBC, triple-negative breast cancer.

^a^ *P* values were calculated using analysis of variance, Kruskal-Wallis, Pearson χ^2^, or Fisher exact tests, as appropriate.

^b^ Distance from residence to hospital was calculated by taking the differences of coordinates (longitudes/latitudes) between the patient’s address and the University of Chicago Medicine Comprehensive Cancer Center’s address, based on the Haversine formula.

^c^ The Area Deprivation Index (national ranking percentile) is a composite measure of income, education, employment, and housing quality that ranks neighborhoods by socioeconomic disadvantage at the national level. It is scored from 1 to 100, with higher scores representing greater neighborhood socioeconomic deprivation.

### Patterns in Discussion of Clinical Trials With Patients

Among survey responders, there were statistically significant differences among all racial and ethnic groups of whether or not the participants had a discussion about a clinical trial with their health care practitioners. The difference between Black patients and White patients was statistically significant, with increased portion of Black patients (104 patients [46.4%]) than White patients (309 patients [36.9%]) having a clinical trial discussion (*P* = .009) (eTable 1 in Supplement 1). This may be a reflection of our catchment area which has a significant proportion of Black women (eFigure in Supplement 1). The unadjusted odds ratio of clinical trial discussion comparing Black patients with White patients was 1.48 (95% CI, 1.10-2.00). However, this difference between Black and White patients was no longer statistically significant when adjusting for clinicopathologic and socioeconomic factors (AOR, 1.31; 95% CI, 0.78-2.21) (Table 2). There was also no difference in trial discussion between Asian patients and White patients (AOR, 0.75; 95% CI, 0.31-1.82) or between Hispanic patients and White patients (AOR, 0.73; 95% CI, 0.26-2.08). There were no significant differences in the distribution of trial participation after clinical trial discussion among all racial and ethnic groups (8 Asian patients [44.4%]; 62 Black patients [60.8%]; 11 Hispanic patients [73.3%]; 204 White patients [66.2%]). In the adjusted model, there were no significant differences between Black patients and White patients (AOR, 0.78; 95% CI, 0.40-1.54), between Hispanic patients and White patients (AOR, 0.51; 95% CI, 0.10-2.72), or between Asian patients and White patients (AOR, 0.37; 95% CI, 0.11-1.26) (Table 2).

**Table 2.** Racial and Ethnic Differences in Clinical Trial Discussion or Participation Among Patients With Breast Cancer

Characteristic	Patients, No. (%)		*P* value^a^	Logistic regression (95% CI)		
	No	Yes		Crude OR	AOR^b^	AOR^c^
**Discussed a breast cancer treatment clinical trial with a health care practitioner (n = 1150)**						
Overall	703 (61.1)	447 (38.9)	NA	NA	NA	NA
Race and ethnicity						
Asian	32 (62.8)	19 (37.2)	.07	1.02 (0.57-1.82)	1.02 (0.51-2.02)	0.75 (0.31-1.82)
Black	120 (53.6)	104 (46.4)		1.48 (1.10-2.00)	1.33 (0.92-1.92)	1.31 (0.78-2.21)
Hispanic	20 (57.1)	15 (42.9)		1.28 (0.65-2.54)	1.38 (0.62-3.06)	0.73 (0.26-2.08)
White	529 (63.1)	309 (36.9)		1 [Reference]	1 [Reference]	1 [Reference]
**Participated in the clinical trial being offered (n = 443)**						
Overall	158 (35.7)	285 (64.3)	NA	NA	NA	NA
Race and ethnicity						
Asian	10 (55.6)	8 (44.4)	.20	0.30 (0.11-0.85)	0.27 (0.09-0.80)	0.37 (0.11-1.26)^d^
Black	40 (39.2)	62 (60.8)		0.77 (0.46-1.30)	0.68 (0.40-1.17)	0.78 (0.40-1.54)^d^
Hispanic	4 (26.7)	11 (73.3)		1.13 (0.34-3.76)	1.14 (0.34-3.82)	0.51 (0.10-2.72)^d^
White	104 (33.8)	204 (66.2)		1 [Reference]	1 [Reference]	1 [Reference]

Abbreviations: AOR, adjusted odds ratio; AJCC, American Joint Committee on Cancer; NA, not applicable; OR, odds ratio.

^a^ *P* values were calculated using Pearson χ^2^ tests.

^b^ Adjusted for AJCC stage and molecular subtype.

^c^ Adjusted for AJCC stage, molecular subtype, Charlson comorbidity index, age at diagnosis, level of education, marital status, type of health insurance, and annual household income.

^d^ Adjusted for AJCC stage, molecular subtype, Charlson comorbidity index, and annual household income.

In analysis of patients who did or did not get offered a clinical trial, we found that patients who were offered a trial were younger, were enrolled in ChiMEC within the last 5 years, had a higher stage, were more likely to be diagnosed with TNBC, and had a high-grade tumor (eTable 2 in Supplement 1). Among 443 patients who were offered a clinical trial, 285 (64.3%) chose to participate while 158 (35.7%) chose to decline (eTable 3 in Supplement 1). There was a significant association between annual household income and clinical trial participation, but the association was not monotonic. Compared with patients with an annual household income of $150 000 or more (56 patients [75.5%]), those who made less than $50 000 (30 patients [65.0%]), $50 000 to $74.999 (30 patients [73.2%]), $75 000 to $99 999 (22 patients [48.9%]), or $100 000 to $149 999 (30 patients [60.0%]) were less likely to have participated in a clinical trial offered (*P* = .03). The proportions of clinical trial participation were similar regardless of distance from residence to hospital and ADI quartiles (eTable 3 in Supplement 1). Patients who specifically came for evaluation for a specific clinical trial were younger, more likely to have high-grade tumors and TNBC, and more likely to be enrolled in Medicaid (eTable 4 in Supplement 1).

### Patient Attitudes Toward Clinical Trial Participation

Patients were then asked their reasons for participating in a clinical trial (Table 3). The most common reasons for trial enrollment was the desire to further research efforts in oncology (229 patients [80.4%]), followed by being aware of the benefits of clinical trial participation (159 patients [55.8%]), wanting to receive the newest therapy (84 patients [29.5%]), and avoiding the adverse effects of standard therapy (16 patients [5.6%]). Asian patients (2 patients [25.0%]) and Hispanic patients (3 patients [27.3%]) were more likely than Black patients (5 patients [8.1%]) and White patients (6 patients [2.9%]) to want to avoid adverse effects as a reason for clinical trial participation (*P* = .001); there were no racial and ethnic differences in other reasons for clinical trial participation (Table 3).

**Table 3.** Patients With Breast Cancer Who Reported Reasons Participated in Clinical Trials, Overall and by Race and Ethnicity

Reason	No. (%)					*P* value^a^
	Overall (n = 285)	Asian (n = 8 [2.8%])	Black (n = 62 [21.8%])	Hispanic (n = 11 [3.9%])	White (n = 204 [71.6%])	
**I was aware of the benefits of participating in clinical trials**						
No	126 (44.2)	1 (12.5)	29 (46.8)	4 (36.4)	92 (45.1)	.31
Yes	159 (55.8)	7 (87.5)	33 (53.2)	7 (63.6)	112 (54.9)	
**I wanted to receive the newest therapy**						
No	201 (70.5)	4 (50.0)	48 (77.4)	8 (72.7)	141 (69.1)	.35
Yes	84 (29.5)	4 (50.0)	14 (22.6)	3 (27.3)	63 (30.9)	
**I wanted to help further research efforts in oncology**						
No	56 (19.7)	3 (37.5)	16 (25.8)	1 (9.1)	36 (17.7)	.21
Yes	229 (80.4)	5 (62.5)	46 (74.2)	10 (90.9)	168 (82.4)	
**I wanted to avoid side effects of standard therapy**						
No	269 (94.4)	6 (75.0)	57 (91.9)	8 (72.7)	198 (97.1)	.001
Yes	16 (5.6)	2 (25.0)	5 (8.1)	3 (27.3)	6 (2.9)	

^a^ *P* values were calculated using Fisher exact test.

Similarly, patients who were offered trials but declined participation were asked their reasons not participating; 158 patients (35.7%) participated in this portion of the survey (Table 4). The 2 most common reasons were ineligibility (37 patients [23.4%]) and lack of interest in trials (37 patients [23.4%]), followed by worry about the potential of getting a placebo (17 patients [10.8%]), worry about the extra time the trial would require (16 patients [10.1%]), worry about delay in starting treatment (14 patients [8.9%]), worry about adverse effects (14 patients [8.9%]), too many additional visits (10 patients [6.3%]), taking too much time from work (7 patients [4.4%]), and too many additional required laboratory tests or biopsies (6 patients [3.8%]). Only 13 patients (8.2%) overall reported declining because they were not aware of the benefits of participation; this was not significant different across racial and ethnic groups (Table 4).

**Table 4.** Patients With Breast Cancer Who Reported Reasons Not to Participate in Clinical Trials, Overall and by Race and Ethnicity

Reason	Patients, No. (%)					*P* value^a^
	Overall (n = 158)	Asian (n = 10 [6.3%])	Black (n = 40 [25.3%])	Hispanic (n = 4 [2.5%])	White (n = 104 [65.8%])	
**Was told that I was not eligible**						
No	121 (76.6)	9 (90.0)	35 (87.5)	1 (25.0)	76 (73.1)	.02
Yes	37 (23.4)	1 (10.0)	5 (12.5)	3 (75.0)	28 (26.9)	
**Too many additional visits**						
No	148 (93.7)	9 (90.0)	38 (95.0)	4 (100)	97 (93.3)	.79
Yes	10 (6.3)	1 (10.0)	2 (5.0)	0	7 (6.7)	
**I did not have or want to spend the extra time the trial would require**						
No	142 (89.9)	8 (80.0)	38 (95.0)	4 (100)	92 (88.5)	.36
Yes	16 (10.1)	2 (20.0)	2 (5.0)	0	12 (11.5)	
**It would take too much time away from work**						
No	151 (95.6)	10 (100)	37 (92.5)	4 (100)	100 (96.1)	.69
Yes	7 (4.4)	0	3 (7.5)	0	4 (3.9)	
**Too many additional laboratory tests or additional biopsies were required**						
No	152 (96.2)	10 (100)	39 (97.5)	4 (100)	99 (95.2)	>.99
Yes	6 (3.8)	0	1 (2.5)	0	5 (4.8)	
**Unaware of the benefits of participation**						
No	145 (91.8)	9 (90.0)	35 (87.5)	4 (100)	97 (93.3)	.58
Yes	13 (8.2)	1 (10.0)	5 (12.5)	0	7 (6.7)	
**Fear of possible side effects**						
No	144 (91.1)	9 (90.0)	36 (90.0)	4 (100)	95 (91.4)	.93
Yes	14 (8.9)	1 (10.0)	4 (10.0)	0	9 (8.7)	
**Worried that it would delay starting treatment**						
No	144 (91.1)	9 (90.0)	36 (90.0)	4 (100)	95 (91.4)	.93
Yes	14 (8.9)	1 (10.0)	4 (10.0)	0	9 (8.7)	
**Worried about the potential of getting a placebo**						
No	141 (89.2)	10 (100)	37 (92.5)	3 (75.0)	91 (87.5)	.39
Yes	17 (10.8)	0	3 (7.5)	1 (25.0)	13 (12.5)	
**Not interested in trials**						
No	121 (76.6)	9 (90.0)	25 (62.5)	4 (100)	83 (79.8)	.08
Yes	37 (23.4)	1 (10.0)	15 (37.5)	0	21 (20.2)	
**Other reasons**						
No	128 (87.1)	6 (60.0)	31 (83.8)	4 (100)	87 (90.6)	.05
Yes	19 (12.9)	4 (40.0)	6 (16.2)	0	9 (9.4)	

^a^ *P* values were calculated using Fisher exact test.

## Discussion

This cross-sectional study evaluated the patterns of oncologist-directed clinical trial discussion and patient attitudes toward clinical trial participation in our diverse patient cohort. Participation of underserved populations in clinical trials is vitally important to both provide equitable care to patients with cancer and to ensure trials are representative of the overall cancer population.^10^ There are numerous levels of barriers and biases to clinical trial participation that include systemic, institutional, clinician-based, and patient-based. The catchment area of our cancer center includes the city of Chicago, Illinois; greater suburbs of Chicago; and areas of Northwest Indiana. Census data of our catchment area showcase a community that is 8% Asian, 19% Black, 22% Hispanic, and 49% White.^13^ This study evaluated clinician and patient-based barriers to racial and ethnic minority clinical trial discussion and participation, addressing knowledge gaps in patient-clinician discussion and clinical trial participation patterns. Our findings also help better understand the patient-reported barriers to clinical trial participation faced by patients from racial and ethnic minority backgrounds, aiming to improve the enrollment of more diverse populations of patients with breast cancer in clinical trials.

Our study found that patients with breast cancer who were younger, had more advanced disease, diagnosed with TNBC, or diagnosed with a high grade tumor were more likely to have been offered clinical trials. An increased proportion of Black patients reported being offered a clinical trial compared with White patients, which is likely a reflection of the unique expertise and clinical trial portfolio in our center favoring TNBC due to the significant proportion of Black women in our catchment area. Interestingly, among patients who were offered a clinical trial, there were no differences between racial and ethnic groups of willingness to participate. This is consistent with other studies in the literature citing similar Black and Hispanic patient participation in clinical trials when offered.^14,15^ Our study also found that Asian patients were less likely to participate in clinical trials when offered compared with White patients in the clinical factor–adjusted model; however, this difference was no longer significant after additionally controlling for socioeconomic indicators. This finding suggests socioeconomic factors may influence clinical trial participation but must be interpreted with caution, given the limited sample size. Tailoring of clinical trial offerings to disease subtypes more likely seen in patients from racial and ethnic minority groups can improve their participation rates of clinical trials.

In this study, we found numerous barriers to clinical trial participation. Nearly 25% of patients in our cohort reported ineligibility as a reason they did not participate, most commonly in Hispanic patients, underscoring the importance of broadening eligibility to increase diversity.^16^ Moreover, financial toxicity of clinical trials is well documented in the literature,^17^ and patients with lower annual household income may be less likely to enroll in clinical trials across multiple cancer types, including breast cancer.^18^ In our cohort, we found that the percentage of clinical trial participation was significantly higher among patients with an annual household income of at least $150 000. However, the association between annual household income and clinical trial participation was not monotonic. Household income alone is overly simplistic to evaluate the complexity of financial toxicity for patients, and more nuanced analyses are needed to better understand financial barriers in future studies. Additional indicators, such as flexibility of work schedules, caregiver burden, and transportation access, are helpful to further refine how socioeconomic factors affect patient barriers to enrollment. Hidden costs and personal time constraints can influence participation independently of income. Lastly, time toxicity was also noted as a concern in our patient cohort, with patients reporting too many visits, taking time away from work, or spending extra time on the trial was a reason to decline participation. Future research is needed to explore the confluence of financial and time toxicities and additional socioeconomic indicators on clinical trial discussion and enrollment among patients with breast cancer. This provides important feedback for the design of clinical trials to reduce additional visits and streamline study schedules, reconsider strict eligibility criteria that may exclude patients, and include funding for patients to pay for ancillary expenses of trial participation.

### Limitations

This study benefits from a large, multiethnic cohort that enabled evaluation of clinician and patient barriers to clinical trial discussion and participation, although not without limitations. One major limitation is that patients who participated in the study may not be fully representative of the breast cancer patient population in the US. Our study sample included patients who were enrolled in a cohort study and actually responded to this study survey, suggesting this sample was a motivated patient population. Second, our analysis may not have sufficient power to detect significance in clinical trial discussion and participation comparing Asian or Hispanic patients with White patients due to limited sample size; however, it did offer insights into the expansion of and equal access to clinical trials among patients with breast cancer. Third, data were collected via surveys, and patients who responded may have been healthier or had a positive experience with clinical trial discussion or participation. Furthermore, respondents likely had increased engagement and/or health literacy, and were more likely to discuss and participate in research overall compared with the general population of patients with breast cancer. Similarly, patients may have recall bias regarding their care and more likely to respond if a clinical trial was a significant part of their care. Additionally, implicit bias in clinician discussion of clinical trials with patients is a recognized barrier to enrollment; however, it was not captured in this survey. Therefore, the proportion of patients who were offered or participated in a clinical trial is likely an overestimate.

## Conclusions

This cross-sectional study of racially and ethnically diverse patients with breast cancer described patient attitudes and barriers to clinical trial participation. Across our cohort, Black patients were most likely to have a clinical trial discussion with a health care practitioner. This is likely related to our center’s focus on trials for TNBC and/or advanced disease, diagnoses that occur with greater frequency in Black patients. Consistent with other studies, we demonstrated that when offered, Black patients were equally as likely to participate in a clinical trial as their White counterparts. These data provide valuable insights that can serve as a roadmap for how to expand access to clinical trials to ensure inclusion of individuals from underrepresented racial and ethnic groups. Reducing barriers to enrollment into clinical trials is a scientific and ethical imperative for the cancer community. Patient-directed strategies, including dedicated philanthropic funds for miscellaneous study costs and development of research networks to promote equitable clinical trial access across multiple institutions, can reduce financial and time toxicity barriers to clinical trial enrollment. These initiatives and other work are ongoing to further improve patient-provide discussion and enhance clinical trial participation for our diverse patients.

## Notes

Supplement 1. **eTable 1.** Racial Differences in Clinical Trial Discussion or Participation Among Patients With Breast Cancer **eTable 2.** Demographic and Clinical Characteristics of Patients With Breast Cancer by Cancer Treatment Clinical Trial Discussion With a Health Care Practitioner **eTable 3.** Demographic and Clinical Characteristics of Patients With Breast Cancer by Clinical Trial Participation **eTable 4.** Demographic and Clinical Characteristics of Patients With Breast Cancer by Goal of Clinical Trial Participation **eFigure.** Distributions of Racial/Ethnic Groups Comparing the Catchment Area and the Study Sample

Supplement 2.
Data Sharing Statement
