# Supplementary material for: Clinical Trial Discussion and Participation in a Breast Cancer Cohort by Race and Ethnicity
Source: JAMA Netw Open. 2025 Jun 12;8(6):e2515205. doi: 10.1001/jamanetworkopen.2025.15205 (PMC12163658; doi:10.1001/jamanetworkopen.2025.15205)
Supplement: Supplement 1. — eTable 1. Racial Differences in Clinical Trial Discussion or Participation Among Patients With Breast Cancer eTable 2. Demographic and Clinical Characteristics of Patients With Breast Cancer by Cancer Treatment Clinical Trial Discussion With a Health Care Practitioner eTable 3. Demographic and Clinical Characteristics of Patients With Breast Cancer by Clinical Trial Participation eTable 4. Demographic and Clinical Characteristics of Patients With Breast Cancer by Goal of Clinical Trial Participation eFigure. Distributions of Racial/Ethnic Groups Comparing the Catchment Area and the Study Sample [file jamanetwopen-e2515205-s001.pdf]

Supplemental Online Content

Chen N, Freeman JQ, Zhao F, et al. Clinical trial discussion and participation in a breast cancer cohort by race and ethnicity . *JAMA Netw Open*. 2025;8(6):e2515205.
doi:10.1001/jamanetworkopen.2025.15205

eTable 1. Racial Differences in Clinical Trial Discussion or Participation Among Patients With Breast Cancer

eTable 2. Demographic and Clinical Characteristics of Patients With Breast Cancer by Cancer Treatment Clinical Trial Discussion With a Health Care Practitioner

eTable 3. Demographic and Clinical Characteristics of Patients With Breast Cancer by Clinical Trial Participation

eTable 4. Demographic and Clinical Characteristics of Patients With Breast Cancer by Goal of Clinical Trial Participation

eFigure. Distributions of Racial/Ethnic Groups Comparing the Catchment Area and the Study Sample

This supplemental material has been provided by the authors to give readers additional information about their work.

© 2025 Chen N et al. *JAMA Network Open*.

eTable 1. Racial Differences in Clinical Trial Discussion or Participation among Patients with Breast Cancer

	**Discussed a breast cancer treatment clinical trial with a provider (N = 1150)**			**Logistic regression**		
	**No**	**Yes**				
**Characteristic**	**703 (61.1%)**	**447 (38.9%)**				
	**No. (%)**	**No. (%)**	***P* value [a]**	**Crude OR (95% CI)**	**AOR [b] (95% CI)**	**AOR [c] (95% CI)**
**Race**						
Black	120 (53.6)	104 (46.4)	.009	1.48 (1.10-2.00)	1.34 (0.92-1.93)	1.25 (0.74-2.12)
White	529 (63.1)	309 (36.9)		1 [Reference]	1 [Reference]	1 [Reference]
	**Participated in the clinical trial being offered (N = 443)**					
	**No**	**Yes**				
	**158 (35.7%)**	**285 (64.3%)**				
	**No. (%)**	**No. (%)**	***P* value [a]**	**Crude OR (95% CI)**	**AOR [b] (95% CI)**	**AOR [d] (95% CI)**
**Race**						
Black	40 (39.2)	62 (60.8)	.32	0.77 (0.46-1.30)	0.69 (0.40-1.19)	0.79 (0.40-1.56)
White	104 (33.8)	204 (66.2)		1 [Reference]	1 [Reference]	1 [Reference]

Abbreviations: No., number; OR, odds ratio; AOR, adjusted odds ratio; CI, confidence interval.

[a] *P* values were calculated using Pearson's Chi-squared tests.

[b] Adjusted for AJCC stage and molecular subtype.

[c] Adjusted for AJCC stage, molecular subtype, Charlson comorbidity index, age at diagnosis, level of education, marital status, type of health insurance, and annual household income.

[d] Adjusted for AJCC stage, molecular subtype, Charlson comorbidity index, and annual household income

© 2025 Chen N et al. *JAMA Network Open*.

eTable 2. Demographic and Clinical Characteristics of Patients with Breast Cancer by Cancer Treatment Clinical Trial Discussion with a Provider

	**Discussed a breast cancer treatment clinical trial with a provider (N = 1150)**		
	**No**	**Yes**	
**Characteristic**	**703 (61.1%)**	**447 (38.9%)**	***P* value** [a]
	**No. (%)**	**No. (%)**	
**Age at survey**, mean (SD)	62.6 (12.0)	60.4 (11.8)	.005
**Age at diagnosis**, mean (SD)	54.4 (11.9)	52.6 (11.8)	.02
**Age group (years)**			
<40	85 (55.2)	69 (44.8)	.18
40-65	459 (60.7)	297 (39.3)	
>65	131 (64.9)	71 (35.1)	
**Duration between diagnosis and survey (years)**			
Median (IQR)	6.7 (3.6, 11.2)	6.3 (3.6, 10.1)	.20
**Duration between ChiMEC enrollement and survey**			
Median (IQR)	6.4 (3.4, 10.3)	5.8 (3.3, 9.5)	.07
<5 years	259 (57.3)	193 (42.7)	.03
≥5 years	444 (63.6)	254 (36.4)	
**Highest level of education**			
High school/GED or less	65 (57.5)	48 (42.5)	.71
Trade/technical school, or some college	110 (59.8)	74 (40.2)	
Associate’s degree	40 (56.3)	31 (43.7)	
Bachelor’s degree	212 (63.3)	123 (36.7)	
Graduate or professional degree	275 (61.7)	171 (38.3)	
**Marital status**			
Married	460 (62.3)	278 (37.7)	.048
Single or not married	101 (52.6)	91 (47.4)	
Divorced, separated, or widowed	70 (61.4)	44 (38.6)	
**Type of health insurance**			
Private	486 (59.5)	331 (40.5)	.18
Medicaid	28 (57.1)	21 (42.9)	
Medicare	141 (65.9)	73 (34.1)	
Other or unknown	48 (68.6)	22 (31.4)	
**Annual household income**			
<$50,000	76 (65.5)	40 (34.5)	.13
$50,000-$74,999	52 (55.3)	42 (44.7)	
$75,000-$99,999	50 (52.6)	45 (47.4)	
$100,000-$149,999	80 (61.1)	51 (38.9)	
≥$150,000	142 (65.7)	74 (34.3)	
**Distance from residence to hospital (miles)** [b]			
Median (IQR)	19.2 (8.9, 32.3)	20.9 (10.8, 32.4)	.29
**Area Deprivation Index** [c]			

© 2025 Chen N et al. *JAMA Network Open*.

1st quartile	241 (63.9)	136 (361.)	.04
2nd quartile	234 (62.7)	139 (37.3)	
3rd quartile	146 (58.6)	103 (41.4)	
4th quartile	51 (52.6)	46 (47.4)	
**Charlson comorbidity index**			
0	598 (60.7)	387 (39.3)	.68
1	40 (64.5)	22 (35.5)	
≥2	37 (56.9)	28 (43.1)	
**Histologic type**			
Ductal	430 (58.4)	306 (41.6)	.87
Lobular	57 (62.0)	35 (38.0)	
Ducal and lobular	33 (61.1)	21 (38.9)	
Other	20 (55.6)	16 (44.4)	
**AJCC Stage group**			
0	135 (68.9)	61 (31.1)	<.001
I	359 (70.3)	152 (29.7)	
II	123 (45.9)	145 (54.1)	
III	40 (35.7)	72 (64.3)	
IV	6 (60.0)	4 (40.0)	
**Molecular subtype**			
HR+/HER2-	353 (62.2)	215 (37.8)	<.001
HER2+	81 (55.1)	66 (44.9)	
TNBC	61 (43.0)	81 (57.0)	
**Tumor grade**			
1	107 (73.3)	39 (26.7)	<.001
2	301 (64.9)	163 (35.1)	
3	222 (53.0)	197 (47.0)	
**Was your goal in coming to the University of Chicago to participate in a clinical trial?**			
No	687 (63.4)	397 (36.6)	<.001
Yes	8 (14.3)	48 (85.7)	

Abbreviations: No., number; SD, standard deviation; IQR, interquartile range; ChiMEC, Chicago Multiethnic Epidemiologic Breast Cancer Cohort; GED, general educational development; HR, hormone receptors; HER2, human epidermal growth factor receptor 2; TNBC, triple-negative breast cancer.

[a] *P* values were calculated using Student's t, Wilcoxon rank-sum, Pearson's Chi-square, or Fisher's exact tests as appropriate.

[b] Distance from residence to hospital was calculated by taking the differences of coordinates (longitudes/latitudes) between the patient's address and the University of Chicago Medicine Comprehensive Cancer Center's address based on the Haversine formula.

[c] The Area Deprivation Index (national ranking percentile), a composite measure consisting domains of income, education, employment, and housing quality, that ranks neighborhoods by socioeconomic disadvantage at the national level. It is scored from 1 to 100, with higher scores representing greater neighborhood socioeconomic deprivation.

© 2025 Chen N et al. *JAMA Network Open*.

eTable 3. Demographic and Clinical Characteristics of Patients with Breast Cancer by Clinical Trial Participation

	**Participated in the clinical trial being offered (N = 443)**		
	**No**	**Yes**	
**Characteristic**	**158 (35.7%)**	**285 (64.3%)**	***P* value** [a]
	**No. (%)**	**No. (%)**	
**Age at survey**, mean (SD)	61.3 (12.3)	60.0 (11.6)	.31
**Age at diagnosis**, mean (SD)	53.7 (12.7)	52.0 (11.3)	.15
**Age group (years)**			
<40	27 (39.1)	42 (60.9)	.25
40-65	96 (32.8)	197 (67.2)	
>65	30 (42.3)	41 (57.7)	
**Duration between diagnosis and survey (years)**			
Median (IQR)	5.7 (3.5, 9.7)	6.4 (3.7, 10.6)	.17
**Duration between ChiMEC enrollement and survey**			
Median (IQR)	5.3 (3.1, 9.0)	6.1 (3.5, 9.7)	.11
<5 years	74 (38.9)	116 (61.1)	.21
≥5 years	84 (33.2)	169 (66.8)	
**Highest level of education**			
High school/GED or less	20 (42.6)	27 (57.4)	.29
Trade/technical school, or some college	33 (44.6)	41 (55.4)	
Associate’s degree	11 (35.5)	20 (64.5)	
Bachelor’s degree	39 (32.0)	83 (68.0)	
Graduate or professional degree	55 (32.5)	114 (67.5)	
**Marital status**			
Married	95 (34.6)	180 (65.4)	.11
Single or not married	30 (33.0)	61 (67.0)	
Divorced, separated, or widowed	22 (50.0)	22 (50.0)	
**Type of health insurance**			
Private	112 (34.3)	215 (65.8)	.61
Medicaid	7 (33.3)	14 (66.7)	
Medicare	31 (42.5)	42 (57.5)	
Other or unknown	8 (36.4)	14 (63.6)	
**Annual household income**			
<$50,000	14 (35.0)	26 (65.0)	.03
$50,000-$74,999	11 (26.8)	30 (73.2)	
$75,000-$99,999	23 (51.1)	22 (48.9)	
$100,000-$149,999	20 (40.0)	30 (60.0)	
≥$150,000	18 (24.3)	56 (75.7)	
**Distance from residence to hospital (miles)** [b]			
Median (IQR)	19.9 (12.1, 27.1)	22.2 (10.0, 34.6)	.13
**Area Deprivation Index** [c]			

© 2025 Chen N et al. *JAMA Network Open*.

1st quartile	39 (28.9)	96 (71.1)	.07
2nd quartile	54 (39.1)	84 (60.9)	
3rd quartile	42 (40.8)	61 (59.2)	
4th quartile	17 (37.8)	28 (62.2)	
**Charlson comorbidity index**			
0	135 (35.3)	248 (64.7)	.81
1	9 (40.9)	13 (59.1)	
≥2	9 (32.1)	19 (67.9)	
**Histologic type**			
Ductal	112 (37.1)	190 (62.9)	.85
Lobular	13 (37.1)	22 (62.9)	
Ducal and lobular	6 (28.6)	15 (71.4)	
Other	5 (31.3)	11 (68.7)	
**AJCC Stage group**			
0	19 (31.2)	42 (68.8)	.96
I	55 (36.7)	95 (63.3)	
II	51 (35.4)	93 (64.6)	
III	25 (34.7)	47 (65.3)	
IV	1 (33.3)	2 (66.7)	
**Molecular subtype**			
HR+/HER2-	85 (39.7)	129 (60.3)	.41
HER2+	23 (34.9)	43 (65.1)	
TNBC	25 (31.7)	54 (68.4)	
**Tumor grade**			
1	9 (23.7)	29 (76.3)	.32
2	59 (36.2)	104 (63.8)	
3	69 (35.6)	125 (64.4)	
**Was your goal in coming to the University of Chicago to participate in a clinical trial?**			
No	152 (38.5)	243 (61.5)	.001
Yes	6 (12.8)	41 (87.2)	

Abbreviations: No., number; SD, standard deviation; IQR, interquartile range; ChiMEC, Chicago Multiethnic Epidemiologic Breast Cancer Cohort; GED, general educational development; HR, hormone receptors; HER2, human epidermal growth factor receptor 2; TNBC, triple-negative breast cancer.

[a] *P* values were calculated using Student's t, Wilcoxon rank-sum, Pearson's Chi-square, or Fisher's exact tests, as appropriate.

[b] Distance from residence to hospital was calculated by taking the differences of coordinates (longitudes/latitudes) between the patient's address and the University of Chicago Medicine Comprehensive Cancer Center's address based on the Haversine formula.

[c] The Area Deprivation Index (national ranking percentile), a composite measure consisting domains of income, education, employment, and housing quality, that ranks neighborhoods by socioeconomic disadvantage at the national level. It is scored from 1 to 100, with higher scores representing greater neighborhood socioeconomic deprivation.

© 2025 Chen N et al. *JAMA Network Open*.

eTable 4. Demographic and Clinical Characteristics of Patients with Breast Cancer by Goal of Clinical Trial Participation

	**Was your goal in coming to the University of Chicago to participate in a clinical trial? (N = 1140)**		
	**No**	**Yes**	
**Characteristic**	**1084 (95.1)**	**56 (4.9%)**	***P* value** [a]
	**No. (%)**	**No. (%)**	
**Age at survey**, mean (SD)	62.0 (11.9)	55.8 (11.8)	<.001
**Age at diagnosis**, mean (SD)	53.9 (11.9)	48.6 (11.6)	.001
**Age group (years)**			
<40	139 (90.3)	15 (9.7)	.005
40-65	715 (95.5)	34 (4.5)	
>65	195 (97.5)	5 (2.5)	
**Duration between diagnosis and survey (years)**			
Median (IQR)	6.4 (3.6, 11.0)	6.7 (3.9, 9.1)	.76
**Duration between ChiMEC enrollement and survey**			
Median (IQR)	6.2 (3.4, 10.1)	6.1 (3.3, 8.7)	.46
<5 years	428 (94.9)	23 (5.1)	.81
≥5 years	656 (95.2)	33 (4.8)	
**Highest level of education**			
High school/GED or less	100 (90.9)	10 (9.1)	.30
Trade/technical school, or some college	175 (95.1)	9 (5.9)	
Associate’s degree	67 (94.4)	4 (5.6)	
Bachelor’s degree	320 (96.1)	13 (3.9)	
Graduate or professional degree	421 (95.5)	20 (4.5)	
**Marital status**			
Married	702 (95.8)	31 (4.2)	.12
Single or not married	178 (92.7)	14 (7.3)	
Divorced, separated, or widowed	107 (97.3)	3 (2.7)	
**Type of health insurance**			
Private	771 (95.2)	39 (4.8)	.006
Medicaid	41 (83.7)	8 (16.3)	
Medicare	206 (97.2)	6 (2.8)	
Other or unknown	66 (95.6)	3 (4.4)	
**Annual household income**			
<$50,000	104 (91.2)	10 (8.8)	.33
$50,000-$74,999	88 (93.6)	6 (6.4)	
$75,000-$99,999	92 (97.9)	2 (2.1)	
$100,000-$149,999	124 (94.7)	7 (5.3)	
≥$150,000	203 (94.9)	11 (5.1)	
**Distance from residence to hospital (miles)** [b]			
Median (IQR)	19.9 (9.8, 32.4)	18.9 (6.6, 31.4)	.54

© 2025 Chen N et al. *JAMA Network Open*.

**Area Deprivation Index** [c]			
1st quartile	350 (93.8)	23 (6.2)	.64
2nd quartile	357 (96.8)	12 (3.3)	
3rd quartile	243 (98.0)	5 (2.0)	
4th quartile	85 (88.5)	11 (11.5)	
**Charlson comorbidity index**			
0	927 (94.9)	50 (5.1)	.59
1	61 (98.4)	1 (1.6)	
≥2	61 (95.3)	3 (4.7)	
**Histologic type**			
Ductal	686 (94.2)	42 (5.8)	.24
Lobular	90 (98.9)	1 (1.1)	
Ducal and lobular	51 (94.4)	3 (5.6)	
Other	35 (97.2)	1 (2.8)	
**AJCC Stage group**			
0	188 (95.9)	8 (4.1)	.07
I	486 (96.2)	19 (3.8)	
II	251 (94.0)	16 (6.0)	
III	103 (92.8)	8 (7.2)	
IV	7 (77.8)	2 (22.2)	
**Molecular subtype**			
HR+/HER2-	548 (97.5)	14 (2.5)	<.001
HER2+	137 (93.8)	9 (6.2)	
TNBC	119 (85.0)	21 (15.0)	
**Tumor grade**			
1	143 (97.9)	3 (2.1)	.001
2	447 (96.7)	15 (3.3)	
3	381 (92.0)	33 (8.0)	

Abbreviations: No, number; SD, standard deviation; IQR, interquartile range; ChiMEC, Chicago Multiethnic Epidemiologic Breast Cancer Cohort; GED, general educational development; HR, hormone receptors; HER2, human epidermal growth factor receptor 2; TNBC, triple-negative breast cancer.

[a] *P* values were calculated using Student's t, Wilcoxon rank-sum, Pearson's Chi-square, or Fisher's exact tests, as appropriate.

[b] Distance from residence to hospital was calculated by taking the differences of coordinates (longitudes/latitudes) between the patient's address and the University of Chicago Medicine Comprehensive Cancer Center's address based on the Haversine formula.

[c] The Area Deprivation Index (national ranking percentile), a composite measure consisting domains of income, education, employment, and housing quality, that ranks neighborhoods by socioeconomic disadvantage at the national level. It is scored from 1 to 100, with higher scores representing greater neighborhood socioeconomic deprivation.

© 2025 Chen N et al. *JAMA Network Open*.

eFigure 1. Distributions of Racial/Ethnic Groups Comparing the Catchment Area and the Study Sample

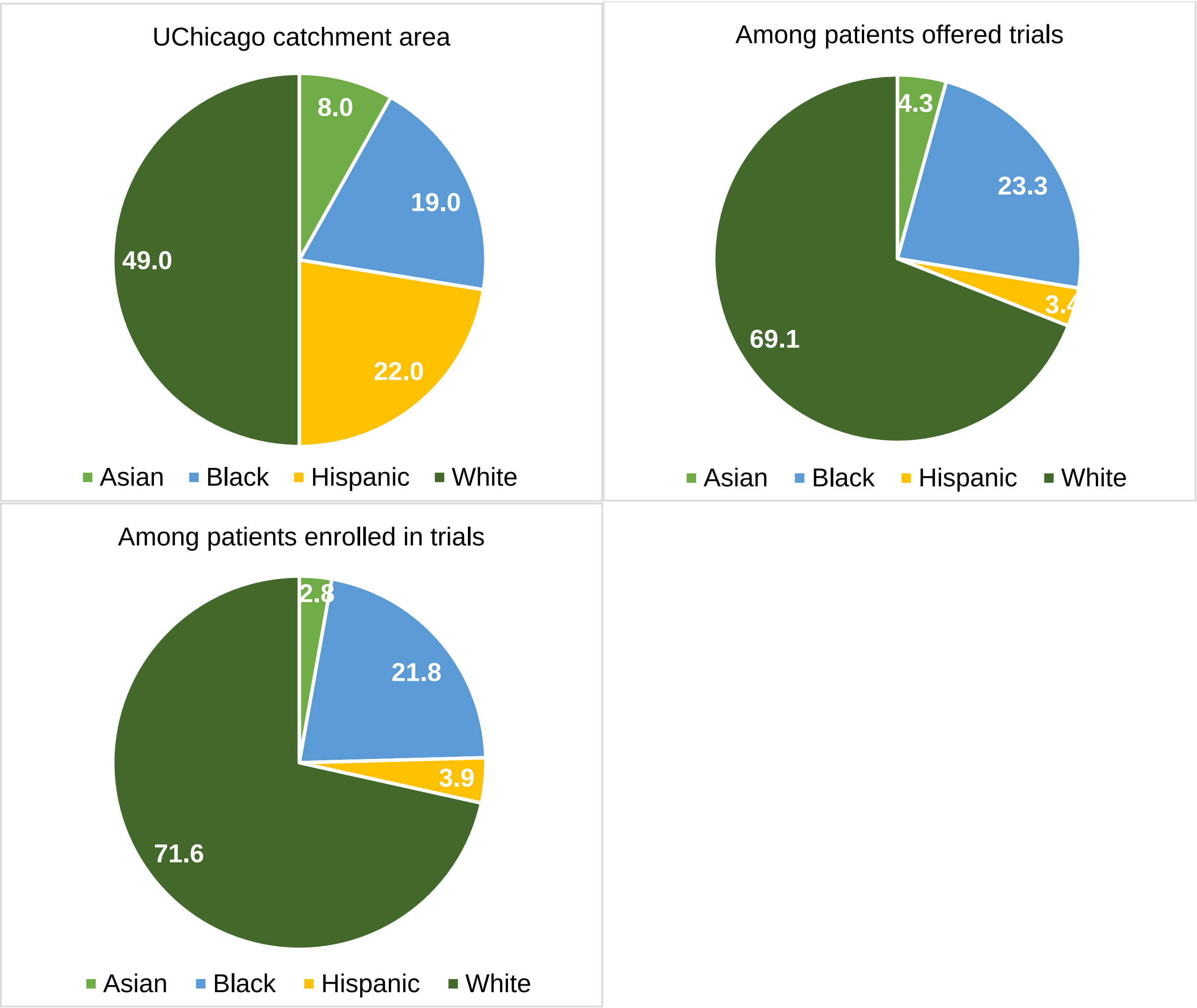

© 2025 Chen N et al. *JAMA Network Open.*
