# Supplementary material for: Clinical Trial Discussion and Participation in a Breast Cancer Cohort by Race and Ethnicity
Source: JAMA Netw Open. 2025 Jun 12;8(6):e2515205. doi: 10.1001/jamanetworkopen.2025.15205 (PMC12163658; doi:10.1001/jamanetworkopen.2025.15205)
Supplement: Supplement 2. — Data Sharing Statement [file jamanetwopen-e2515205-s002.pdf]

Data Sharing Statement

Chen. Clinical Trial Discussion and Participation in a Breast Cancer Cohort by Race and Ethnicity. *JAMA Netw Open*. Published June 12, 2025.
doi:10.1001/jamanetworkopen.2025.15205

Data
Data available: No

Additional Information
Explanation for why data not available: Data for this study cannot be shared publicly due to patient confidentiality and privacy concerns. However, the data can be requested upon reasonable request pending the approval of the University of Chicago Institutional Review Board and the corresponding authors. Supporting Documents Document types: None Additional Information Who can access the data: Anyone requesting the data Types of analyses: Any purpose Mechanisms of data availability: Requires a request to the corresponding authors.
